# Effects of different reclamation practices on cotton root morphological characteristics and yield in coastal saline land

**DOI:** 10.3389/fpls.2025.1680016

**Published:** 2025-12-03

**Authors:** Jingsong Li, Xiaohui Feng, Xiaoguang Li, Xiaojing Liu, Fengcui Fan, Kai Guo

**Affiliations:** 1 CAS Engineering Laboratory for Efficient Utilization of Saline Resources, Center for Agricultural Resources Research, Institute of Genetics and Developmental Biology, Chinese Academy of Sciences, Shijiazhuang, China; 2 The Institute of Agricultural Information and Economics (IAIE), Hebei Academy of Agriculture and Forestry Sciences, Shijiazhuang, China; 3 University of Chinese Academy of Sciences, Beijing, China; 4 Hebei Normal University, Shijiazhuang, China

**Keywords:** cotton, root traits, coastal land, soil salinity, root length density

## Abstract

Plastic mulching (PM), straw interlayer (SI), and organic amendment (OA) have been reported to effectively increase cotton yield in coastal saline lands with dry climates. However, the adaptation of cotton roots to changes in soil physical and chemical properties remains unclear. In this study, a field experiment on rain-fed cotton (*Gossypium hirsutum* L.) under different reclamation practices was conducted in Bohai coastal land, China. Soil structure [mean weight diameter (MWD) and bulk density (BD)], nutrients, soil water, and salt profiles were examined in relation to cotton root distribution, morphological traits [root length density (RLD), root surface area (RSA), root volume (RV), and root average diameter (RAD)], and root diameter proportions. The results showed that PM increased 7.42% soil water content and reduced 52.06% salt content in the 0–10 cm soil. These soil environment changes led to 21.21% increase in RLD, but 16.56% decrease in RAD, mainly due to an increase in the percentage of fine root (diameter < 1.0 mm) from 72.5% [control (CK)] to 83.7%; SI decreased 45.36% topsoil salt content and improved 31.28% cotton yield, but it had no significant impact on root morphological traits; OA significantly improved soil structure (64.37% increase in MWD and 9.56% decrease in BD) and nutrient properties, as well as reduced 25.73% soil salt content. Compared with PM and SI, OA showed greater promotion on RLD (60.61%), RSA (69.57%), and RV (25.37%), but had little influence on RAD. A structural equation model indicated that fine roots contributed to the increase in cotton yield and were promoted by soil water, structure, and nutrients, while being negatively correlated with soil salinity. In contrast, coarse roots (diameter > 1.0 mm) were positively correlated with soil salt content. These findings suggest that cotton plants can mitigate salt stress by optimizing root foraging in the most favorable soil zones, allocating more fine root growth to areas with higher moisture, greater nutrients, better soil structure, and lower salt content. The composition of root diameter was primarily determined by soil water and salt content rather than soil structure or nutrients. This root morphological response to the soil environment is significant for cotton production in coastal saline lands.

## 1 Introduction

Soil salinization is a major global issue affecting agricultural production, with approximately 800 million hectares of soil worldwide experiencing varying degrees of salinization (Keesstra, 2016). Over the past 50 years, water conservancy projects have been widely implemented to leach and drain soil salts, reclaiming large areas of saline land. However, this approach is limited in water-deficient areas, such as the Bohai coastal land in China (Hopmans et al., 2021; Feng et al., 2023). In this region, there are 11.97 million hectares of salt-affected soil, with salt content reaching up to 10 g/kg (Cao et al., 2021). To enhance crop production in saline land, water-saving ameliorations are urgently needed to address both drought and soil salinity issues.

In coastal lands, salts accumulate on the soil surface mainly due to the capillary movement of water rising from the shallow underground water with high salinity (Rose et al., 2005; Skrzypek et al., 2016). Salts not only limit the soil nutrient availability but also degrade soil structure, which adversely affects plant growth (Dendooven et al., 2010; Singh, 2016). Several farming practices have been reported to reduce soil salt content: soil surface mulching with plastic film to reduce the evaporation-induced salt accumulation (Ke et al., 2021; Song et al., 2025), burying a straw layer in the soil to block the upward movement of salts (Zhang et al., 2020; Zhao et al., 2016), and incorporating organic amendments to improve the soil properties (Chen et al., 2022; Yang et al., 2018; Jessica et al., 2015). All of these methods have been effective in enhancing crop production in salt-affected land. Previous studies have explored the soil water and salt transport within the profile (Li et al., 2024b; Umidbek et al., 2024), but knowledge remains limited regarding how plants adapt to the restored soil environment after reclamation.

Cotton (*Gossypium hirsutum* L.) is strongly tolerant to salt stress, with a threshold soil salinity of 4.9 g/kg, which made it one of the most suitable crops for cultivation in saline-alkali soils (Gupta, 1986). Studies on cotton root traits in saline soils are limited compared to shoot traits, primarily due to the inherent difficulties in imaging and quantifying root growth in soil. It is well established that root morphology is significantly influenced by the soil environment. Soil salinity can substantially reduce the cotton yield by inhibiting root elongation and limiting the roots’ ability to absorb water and nutrients (Zhang L. et al., 2014). Furthermore, the study of Wang et al. (2018) demonstrated that the ratio of fine to coarse roots and the spatial distribution of roots within the soil profile play crucial roles in cotton’s ability to resist salt stress. Under saline conditions, cotton allocates more biomass to fine roots to maintain the total root length, thereby enhancing root foraging capacity. In coastal soils with shallow saline groundwater, the most notable root system characteristic of cotton is the inhibition of the primary axial root, accompanied by increased lateral root development in the topsoil. This adaptation is an important strategy to avoid soil salt toxicity. However, the detailed relationships between cotton root morphological growth and coastal soil profiles remain unclear and warrant further investigation.

Thus, a 2-year field experiment was conducted on the rain-fed cotton in the Bohai coastal saline land, China, employing different farming practices, including plastic mulching (PM), straw interlayer (SI), organic amendment (OA), and a control (CK). Soil structure, nutrients, water, and salt profiles, and their relationship with cotton root morphology were examined to explore the interaction between cotton root establishment and soil environment. The results of this study could provide a theoretical basis for cotton production in coastal salt-affected lands.

## 2 Materials and methods

### 2.1 Experimental site

**Figure 1:**
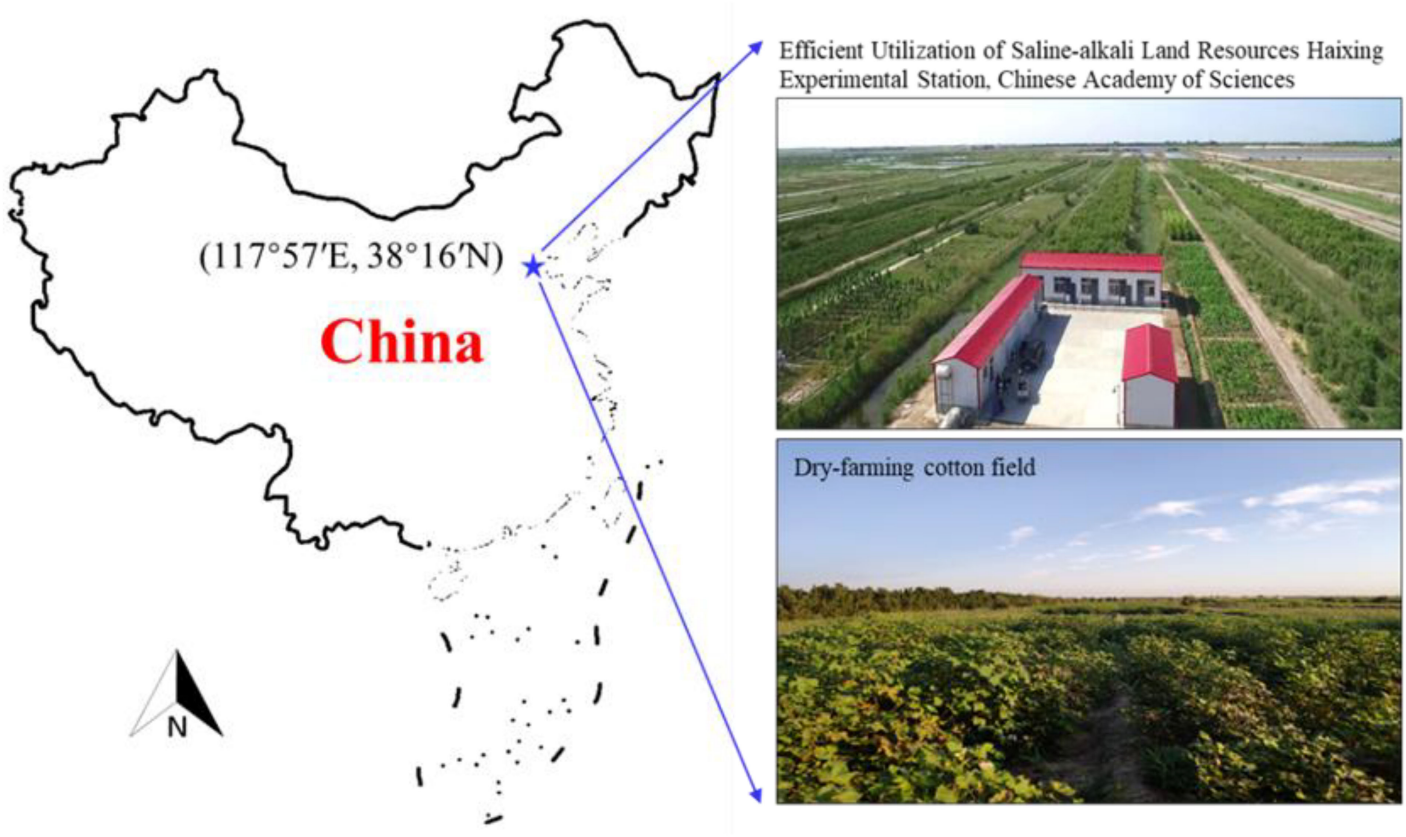
Geographical location of study site and the cotton farming scene.

A field experiment was carried out at the Efficient Utilization of Saline-alkali Land Resources Haixing Experimental Station, Chinese Academy of Sciences, near the Bohai Sea Bay, in Haixing County, Hebei Province, China (117°57′17″–117°58′31″E, 38°16′83″–38°17′59″N) (**Figure 1**). It has a semi-humid continental monsoon climate, characterized by a short rainy summer and long dry spring, autumn, and winter (Guo and Liu, 2015). The annual average precipitation was 582 mm, with more than 75% of the rainfall occurring during July to September (Xia et al., 2019). The elevation of the experimental site is 2.8 m, the groundwater level ranges from 0.8 m to 2.0 m, and the salt content of the groundwater ranges from 10 to 40 g/L (Ju et al., 2019). The soil in this region is highly saline with a silty clay loam texture (clay 2.37%, silt 83.47%, and sand 14.16%). The background of soil nutrient elements is alkali hydrolyzed nitrogen 59.17 mg/kg, available phosphorus 3.08 mg/kg, and available potassium 279.5 mg/kg.

### 2.2 Materials and treatments

**Figure 2:**
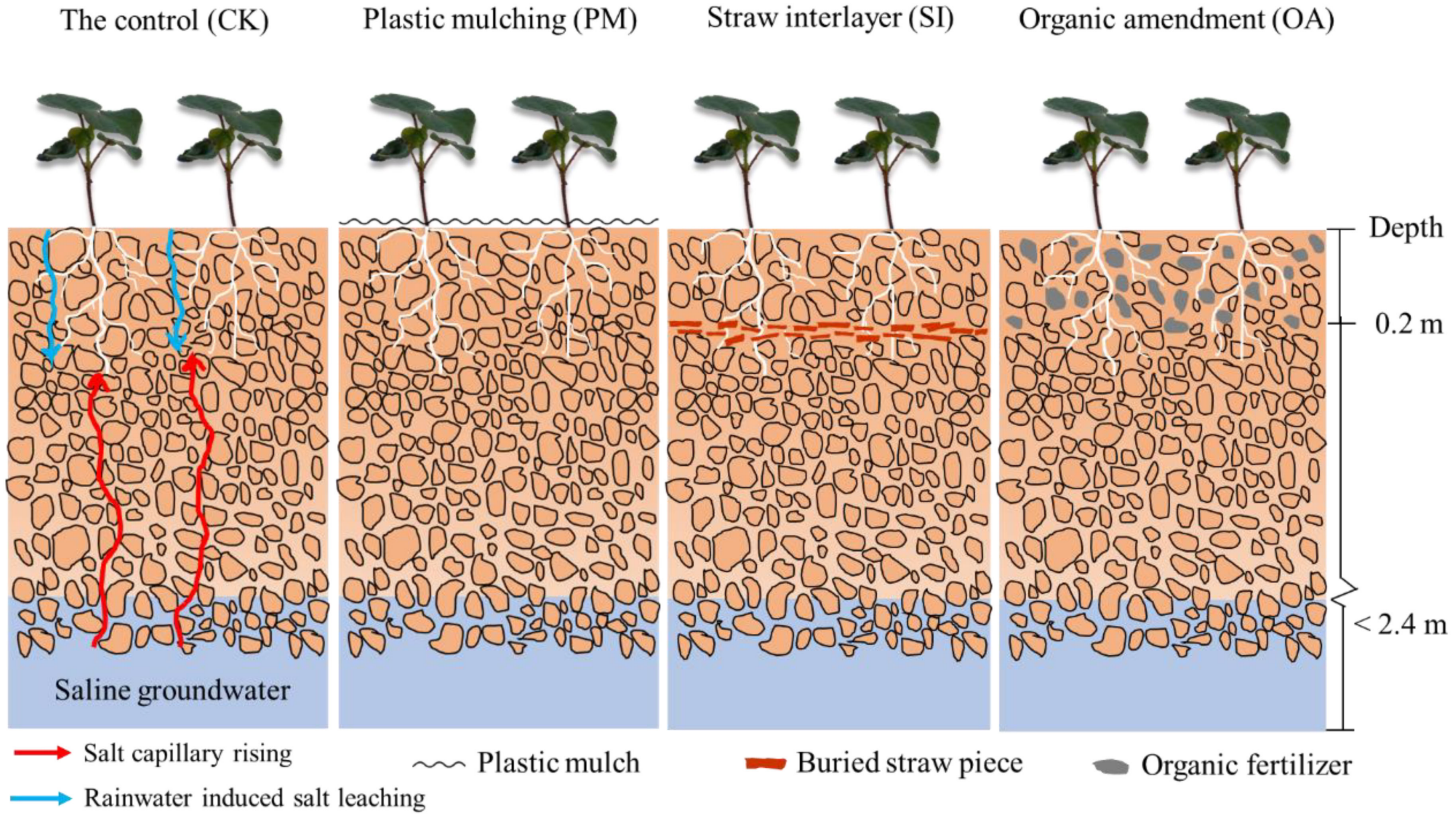
The schematic diagram of soil profile for the control (CK), plastic mulching (PM), straw interlayer (SI), and organic amendment (OA) treatments.

In December 2019, farmland soil was rotary tilled, and treatments were conducted as follows (**Figure 2**). CK: 0–20 cm soil was rotary tilled using a small-type rotocultivator. PM: After rotary tillage, transparent plastic film (0.1-mm thickness) was mulched on the soil surface. SI: Straw pieces (2–3-cm length, air-dried corn straw from the local field) were tilled and buried at 20-cm soil depth, and then the topsoil was backfilled. The straw application amount was 6.0 t/mu (Zhang et al., 2020). OA: Organic fertilizer (49.3% organic matter content; fermented from cereals and corn straw; Hua Yu Agricultural Science and Technology Co., Ltd., Handan, China) was incorporated into the 0–20-cm-depth topsoil during rotary tillage. The organic fertilizer application amount was 3.0 t/mu. The microplot area for the experiment was 5 m × 5 m, and each treatment had four replications.

The cotton (*Gossypium* spp., Luyan Cotton 37) was sown on 7 April and harvested on 25 October 2020, and sown on 9 April and harvested on 29 October 2021. The sowing density of cotton was 3.45 × 10^3^ plant/ha, and N–P_2_O_5_–K_2_O (20%–20%–20%) was used as the base fertilizer at a rate of 3.0 t/ha. No artificial irrigation was conducted during the growth of cotton, and the crop was grown totally under dry-farming conditions.

### 2.3 Soil properties

**Figure 3:**
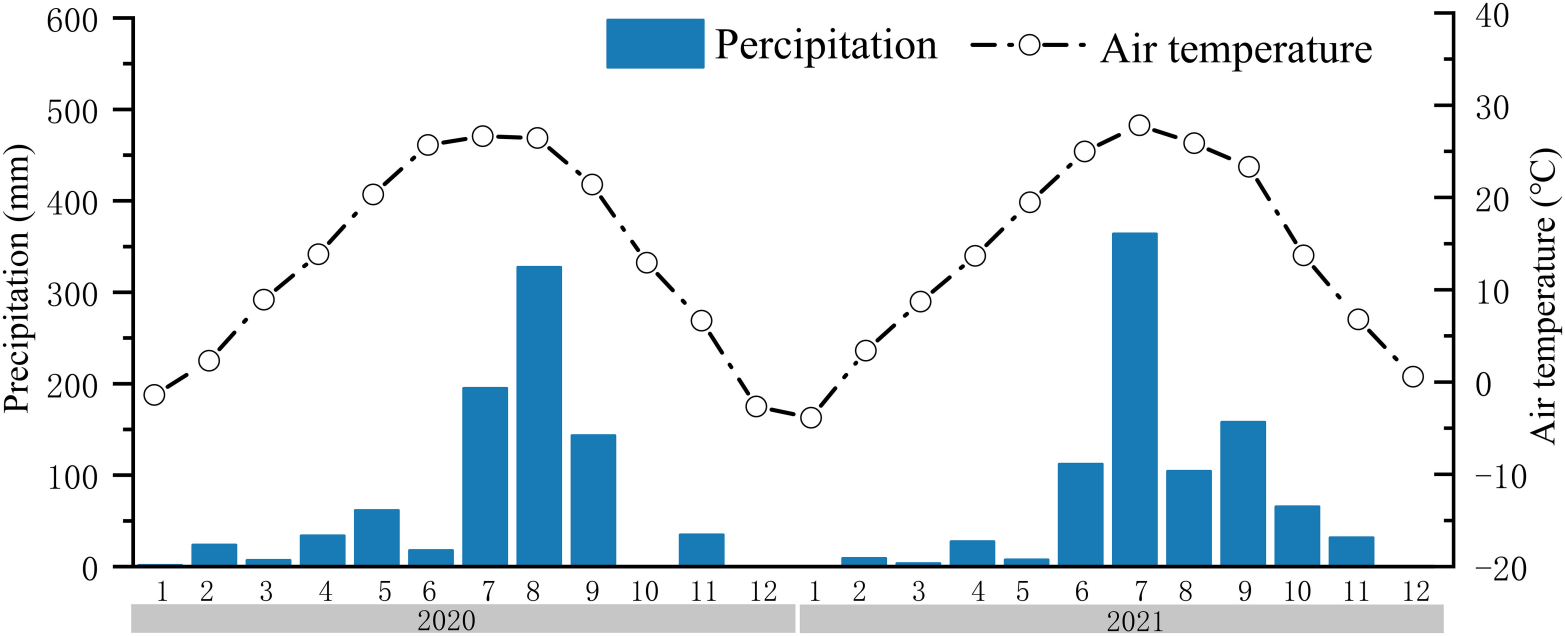
The precipitation and air temperature at study site in 2020 and 2021.

The data of air temperature and precipitation were obtained from a weather station (INSENTEK, China) at the study site (**Figure 3**). According to our previous studies, there is a seasonal soil water and salt dynamics in this region (Li et al., 2024a; Guo and Liu, 2020), and the soil salt content rises to the highest value during spring (March to May). Thus, 27 April 2020 and 15 April 2021 were selected to collect the soil samples, which could mostly reflect the degree of soil salinization. Soil samples were collected at depth intervals of 0.2 to 0.8 m. Steel rings (100 cm^3^) were used to measure soil bulk density (BD) and soil water storage capacity (SWSC). In the laboratory, soil cores were saturated by capillarity for 24 h and then weighed, and the volumetric water content was measured at matric suctions of 30 kPa using the centrifugal method. Finally, the cores were oven-dried at 105 °C for 24 h to measure soil BD. Soil total porosity (TP) was calculated as follows, assuming a soil particle density (ds) of 2.65 g/cm^3^ (Anon, 1982).

(1) TP=(1–BD/ds)∗100

SWSC was calculated as follows:

(2) SWSC=θFC/TP

where θFC is the volumetric water content at the field capacity, which is considered equal to θ_30kPa_ (Cavalcanti et al., 2020).

Soil organic matter (SOM) content was measured using the potassium dichromate method. Undisturbed soil was sampled to measure soil aggregates using the standard wet method described by Kemper and Chepil (1965). Soil mean weight diameter (MWD) was calculated using the following relationships:

(3) $$\text{MWD}=\sum {\text{X}}_{\text{i}}*{\text{W}}_{\text{i}}$$

where X_i_ indicates the mean diameter of each size fraction and W_i_ indicates the proportion of soil aggregate weights in the corresponding size.

To examine the soil water and salt profile, soil samples were collected at depth intervals of 0.1 to 0.6 m during the cotton seedling stage (25 May 2020 and 29 May 2021), when the soil salinity is at the highest value during the whole year. Gravimetric soil water content was measured by weighing the soil sample after being oven-dried at 105°C; soil salt content was calculated as the sum of main salt ions, including Cl^−^, SO_4_^2−^, HCO_3_^−^, Na^+^, K^+^, Ca^2+^, and Mg^2+^, which was measured using the titration method by passing dry soil through a 1-mm sieve (Guo and Liu, 2015).

### 2.4 Cotton growth

Plant samples were collected on 4 July 2020 and 10 July 2021. The aboveground biomass was weighed after oven-drying. As the root length density of cotton reaches its maximum during the flowering period (Yan et al., 2009), a root core (10-cm diameter and 10-cm height) and a dig sampling method were used for root sampling at depth intervals of 10 to 60 cm, during this period in 2021. After cleaning and washing, root samples were scanned and analyzed using the WinRHIZO root analysis system (Regent Instruments Inc., Quebec, Canada) to measure root length density (RLD), root surface area (RSA), root volume (RV), and root average diameter (RAD), following the method described by Liu et al. (2020). After harvest (21 October 2020 and 25 October 2021), air-dried cotton fiber without boll hulls was weighed to determine yield. Each treatment was replicated four times.

### 2.5 Date analysis

The SPSS software (SPSS Inc., Chicago, IL, USA) was used for the analysis of variance (ANOVA). Multiple comparisons of values and significant differences between treatments were determined using the least significant difference (LSD) test at p ≤ 0.05. A correlation plot for the relationships between plant parameters and soil properties was conducted using Pearson’s correlation analysis, with the Origin software (OriginLab Corp., Northampton, MA, USA), using the field experiment data in 2021. Then, structural equation modeling (SEM) was used to examine the direct effects of farming practices on soil environment, cotton root traits, and yield production.

## 3 Results

### 3.1 Changes in soil properties

#### 3.1.1 Soil structure

**Figure 4:**
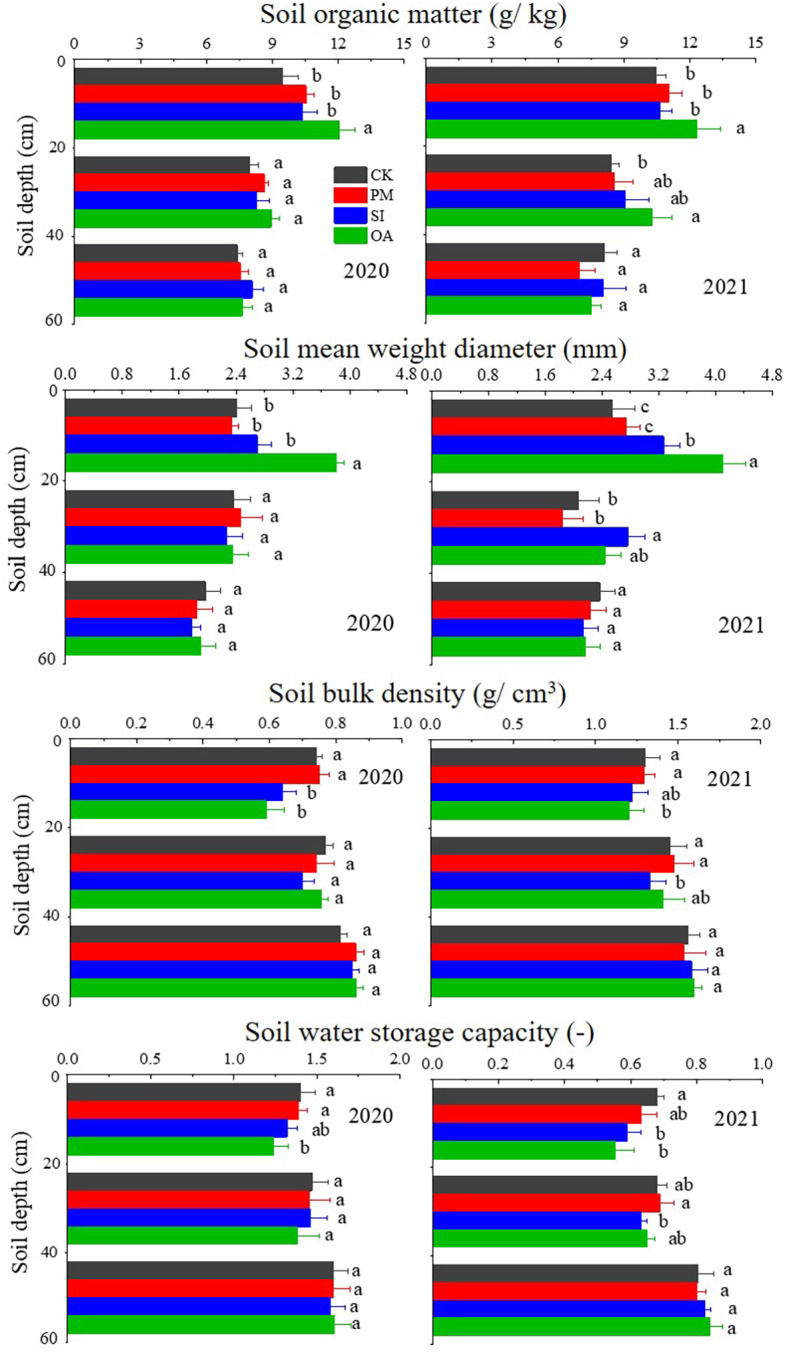
Soil organic matter (SOM), mean weight diameter (MWD), bulk density (BD), and soil water storage capacity (SWSC) under the control (CK), plastic mulching (PM), straw interlayer (SI), and organic amendment (OA) treatments. Note: Values represent means ± SD. Different letters indicate the significant difference in the same soil layer at p ≤ 0.05.

With the increase in soil depth, BD and SWSC (Equations 1 and 2) gradually increased, while SOM and MWD (Equation 3) (**Figure 4**). There was no significant difference between PM and CK on those soil properties in each soil layer. SI also had little impact on SOM and MWD, but reduced 0–20 cm topsoil BD by 6.42% in 2020 and 6.15% in 2021, and significantly limited topsoil SWSC in both years. Compared with PM and SI, OA showed greater promotion effects on topsoil. In 2020, OA significantly improved 27.14% of SOM and 54.27% of MWD in topsoil, which were significantly higher than those under PM and SI. In 2021, not only the topsoil but also the 20–40 cm soil under OA was significantly higher than that under CK. The topsoil BD under OA was 1.24 g/cm^3^ in 2020, 10.79% lower than that under PM (1.39 g/cm^3^) and 6.06% lower than that under SI (1.32 g/cm^3^). The SWSC of OA (0.59 in 2020 and 0.55 in 2021) was also significantly lower than that of CK (0.74 in 2020 and 0.68 in 2021) and PM (0.75 in 2020 and 0.63 in 2021).

#### 3.1.2 Soil nutrient properties

As shown in **Table 1**, OA significantly improved 77.30% of AN, 130.33% of AP, and 63.62% of AK at the 0–20 cm soil layer in 2020, which were higher than those of PM and SI (**Table 1**). In 2021, PM and SI had little influence on AN and AK at 0–20 cm, but significantly improved 89.98% and 65.03% AP, respectively. For the 20–60 cm soil layer, all those treatments had little impact on soil nutrient properties in 2020. In 2021, OA significantly increased the 20–60 cm soil AP by 93.53%.

**Table 1:** Soil alkali hydrolyzed nitrogen (AN), available phosphorus (AP), and available potassium (AK) under the control (CK), plastic mulching (PM), straw interlayer (SI), and organic amendment (OA) treatments.

Soil depth (cm)	Treatment	2020			2021		
		AN (mg/kg)	AP (mg/kg)	AK (mg/kg)	AN (mg/kg)	AP (mg/kg)	AK (mg/kg)
0–20	CK	70.49 ± 14.19 b	3.41 ± 0.82 b	210.44 ± 16.35 b	94.62 ± 9.24 a	2.81 ± 0.42 c	241.75 ± 7.06 b
	PM	81.61 ± 9.22 b	4.32 ± 0.60 b	241.68 ± 20.71 b	85.70 ± 15.11 a	5.33 ± 0.60 b	279.69 ± 12.51 b
	SI	75.36 ± 10.37 b	4.68 ± 0.97 b	256.71 ± 36.92 b	108.69 ± 21.37 a	4.65 ± 0.61 b	235.32 ± 23.64 b
	OA	124.98 ± 24.23 a	7.84 ± 1.02 a	344.32 ± 41.66 a	153.35 ± 30.82 a	9.80 ± 0.89 a	348.54 ± 42.91 a
20–60	CK	54.12 ± 7.43 a	2.66 ± 0.34 a	106.27 ± 10.05 a	50.42 ± 6.29 a	1.39 ± 0.46 b	114.60 ± 8.67 a
	PM	55.92 ± 6.01 a	1.98 ± 0.26 a	154.36 ± 13.18 a	56.36 ± 10.12 a	1.43 ± 0.37 b	145.53 ± 11.38 a
	SI	58.19 ± 7.34 a	2.21 ± 0.49 a	118.66 ± 26.51 a	58.41 ± 6.35 a	2.06 ± 0.21 ab	169.48 ± 25.24 a
	OA	51.34 ± 5.20 a	2.04 ± 0.63 a	139.64 ± 27.45 a	51.93 ± 10.22 a	2.69 ± 0.51 a	187.35 ± 13.97 a

Values represent means ± SD. Different letters indicate the significant difference in the same soil layer at p ≤ 0.05.

#### 3.1.3 Soil water and salt content

**Figure 5:**
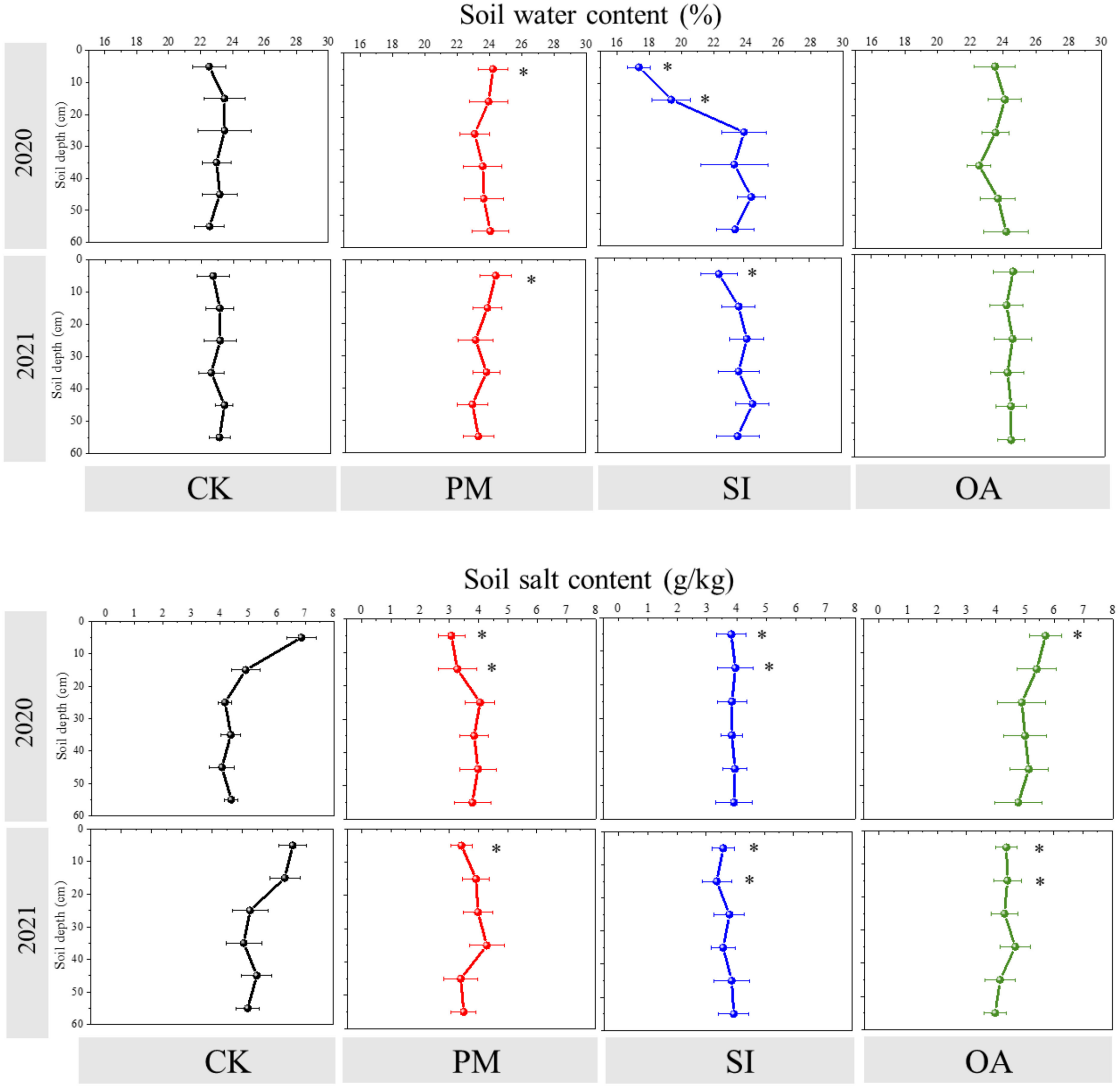
Soil water content (%) and salt content (g/kg) in different soil depths under the control (CK), plastic mulching (PM), straw interlayer (SI), and organic amendment (OA) treatments. Values represent means ± SD. *indicates the significant difference between treatment with CK at p ≤ 0.05.

For CK, there was no difference in soil water content among different soil layers, while soil salt content was observed to decrease with soil depths, and the salt content of the 0–10 cm soil was greatly higher than that of 20–60-cm-deep soil layers (**Figure 5**). Briefly, PM, SI, and OA changed soil water content and salt content mainly in the 0–20 cm soil layer and had little impact on the 20–60-cm-deep soil layer. Compared with those under CK, higher soil water content and lower salt content at the 0–10 cm soil layer were observed under PM in both years. In 2020, soil salt content under PM at 0–10 and 10–20 cm was significantly lower than that under CK at the same soil depth. Differing from the promotional effect of PM on soil water content, SI significantly reduced 17.36% of topsoil water content in 2020 and 22.45% in 2021. Meanwhile, soil salt content at 0–20 cm under SI was observed to be significantly lower than that under CK in 2020 and 2021. OA did not change the topsoil water content but significantly inhibited soil salt content by 16.18% in 2020 and 29.17% in 2021. Combining the 2-year value showed that PM, SI, and OA decreased the 0–10 cm soil salt content by 52.06%, 45.36%, and 25.73%, respectively.

### 3.2 Cotton growth parameters and root morphological traits

#### 3.2.1 Cotton growth

**Figure 6:**
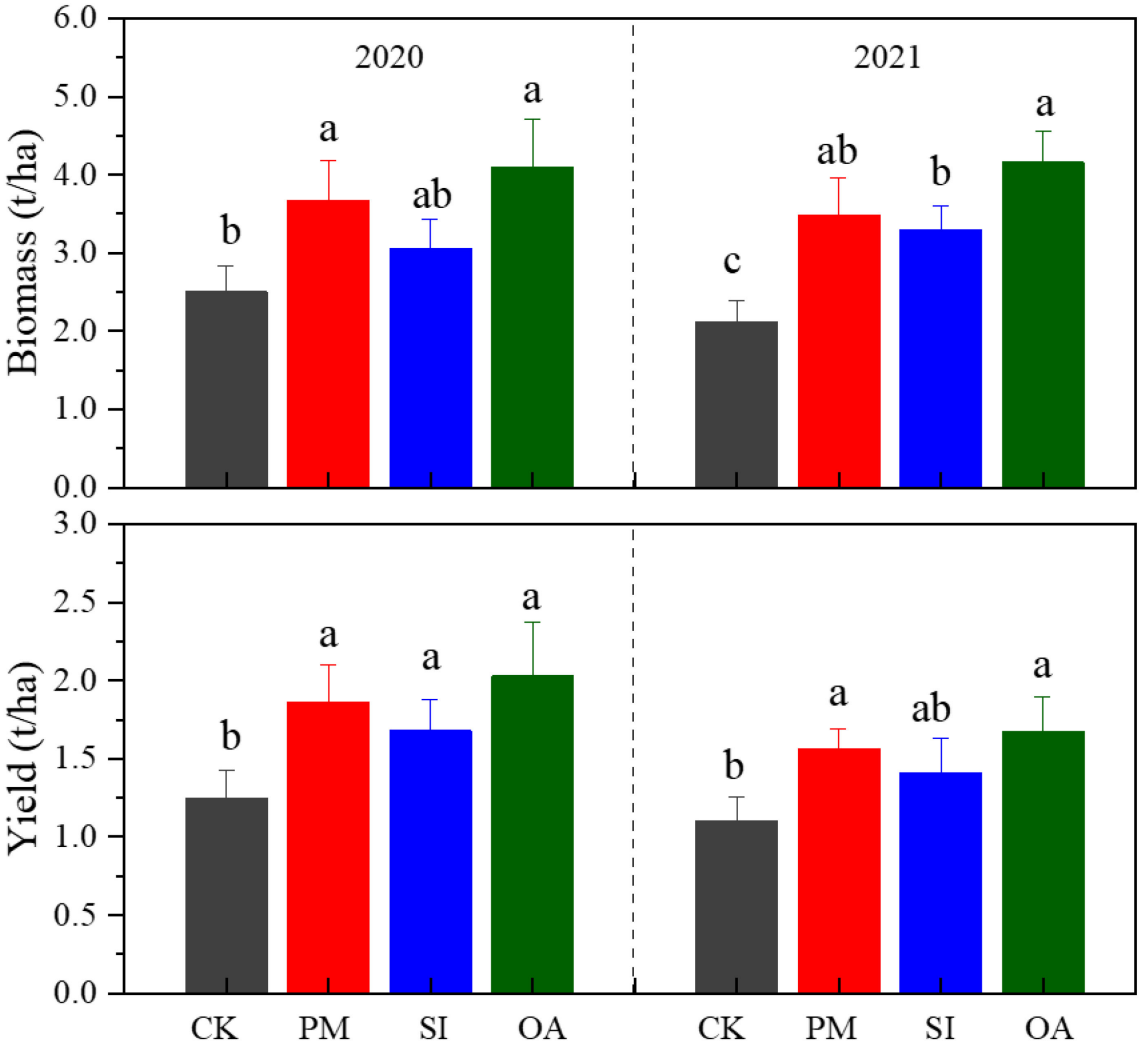
Biomass and yield of cotton under the control (CK), plastic mulching (PM), straw interlayer (SI), and organic amendment (OA). Values represent means ± SD. Different letters indicate the significant difference among treatments at p ≤ 0.05.

PM, SI, and OA all showed positive impacts on the growth of cotton (**Figure 6**). The biomass of cotton under CK was 2.50 t/ha in 2020 and 2.13 t/ha in 2021, which were both significantly lower than those under PM and OA. PM increased biomass by 46.40% in 2020 and 64.4% in 2021, and OA increased that by 63.71% in 2020 and 95.53% in 2021. Even though there was no significant difference in cotton yield between PM and OA, the yield of OA was 9.03% and 6.91% higher than that of PM in 2020 and 2021, respectively. The promotion effect of SI on cotton growth was less than that of PM and OA. Nonetheless, SI promoted a 34.35% yield in 2020 and a 28.21% yield in 2021.

#### 3.2.3 Cotton root morphological traits

**Figure 7:**
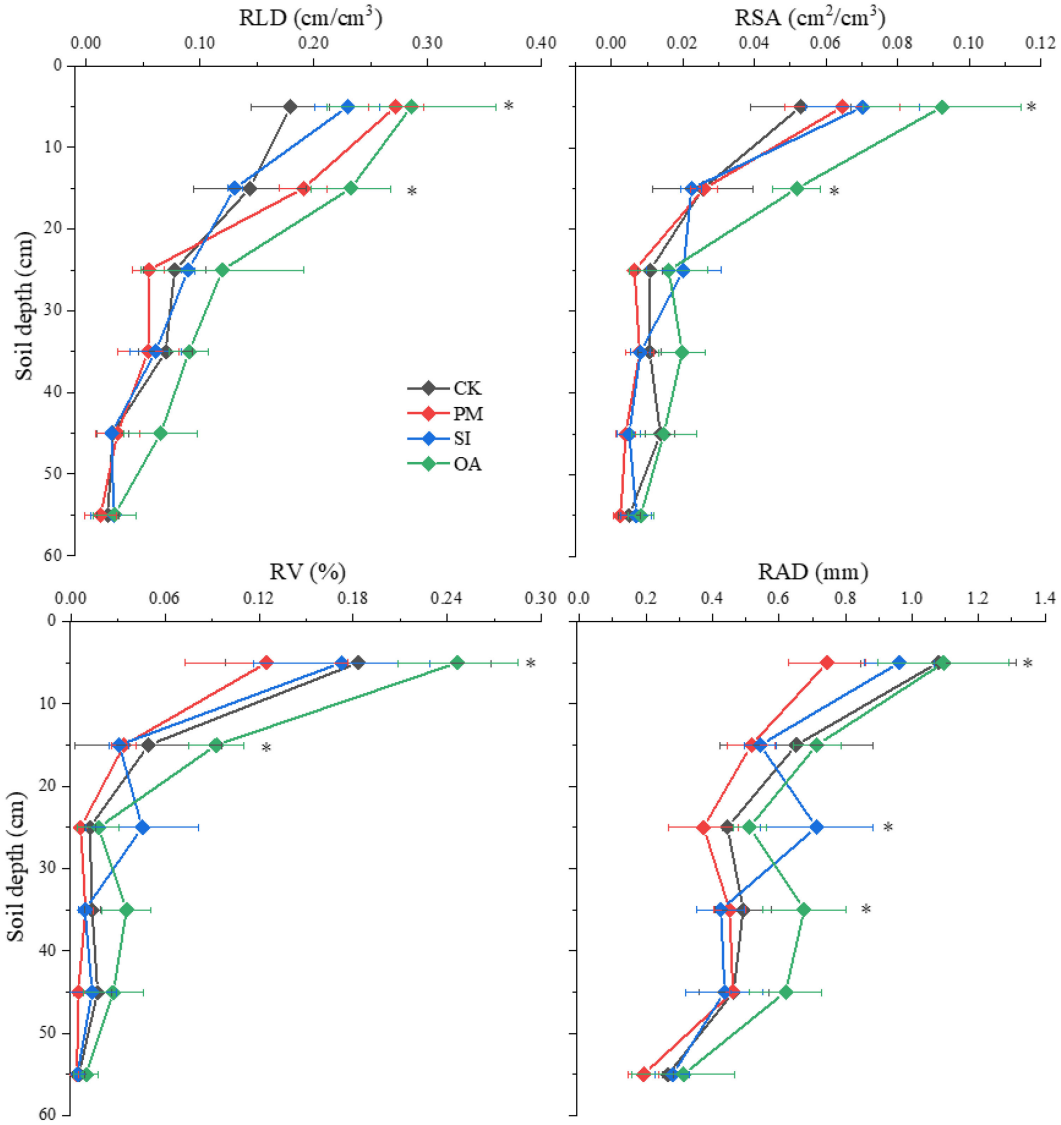
Root length density (RLD), root surface area (RSA), root volume (RV), and root average diameter (RAD) in different soil layers under the control (CK), plastic mulching (PM), straw interlayer (SI), and organic amendment (OA). Values represent means ± SD. *indicates the significant difference between treatment with CK at p ≤ 0.05.

**Figure 8:**
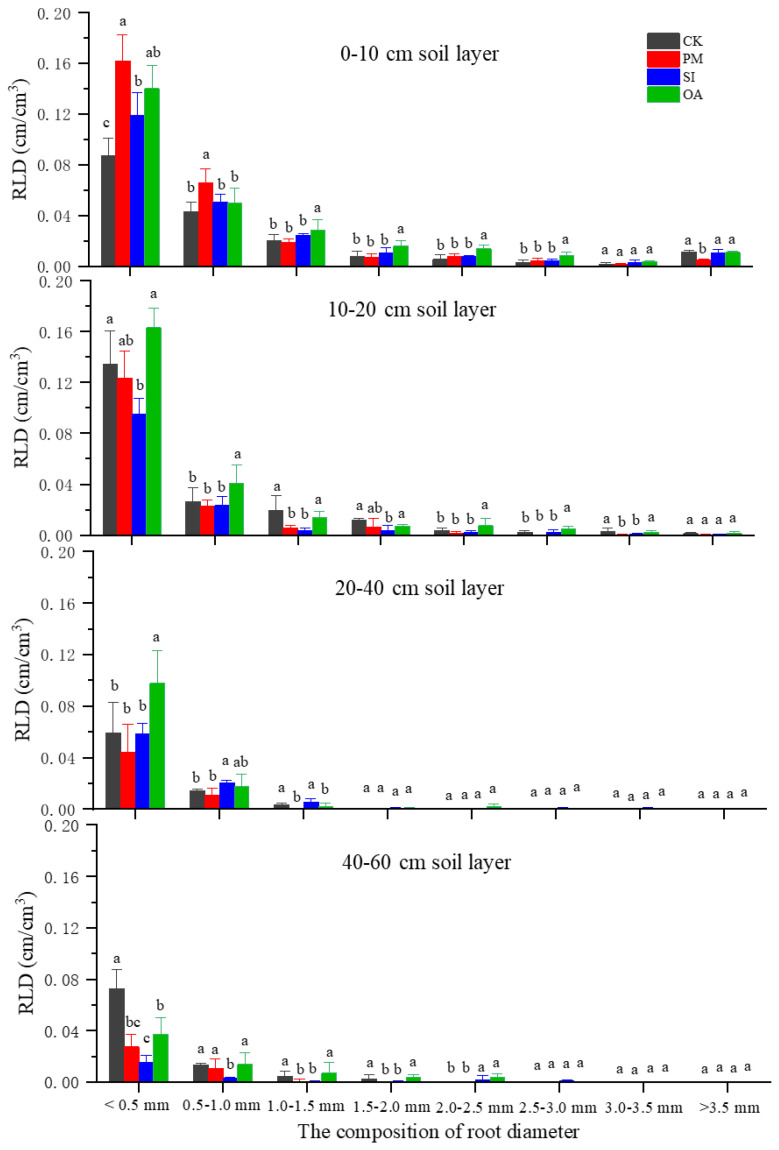
The root length density (RLD) of different root diameters under the control (CK), plastic mulching (PM), straw interlayer (SI), and organic amendment (OA). Note: Values represent means ± SD. Different letters indicate the significant difference among treatments at p ≤ 0.05.

The average RLD under CK was 0.099 cm/cm^3^ (**Table 2**), and the RLD in each soil layer gradually decreased with the increase in soil depth (**Figure 7**). PM significantly improved 21.2% of RLD, but significantly decreased 16.6% of RAD. On the one hand, its promotion effect on RLD was mainly on the root distributed at the topsoil (0–20 cm). For the 20–60 cm soil layer, there was no significant difference between RLD under PM and CK. On the other hand, the percentage of roots with a diameter < 1.0 mm under CK, SI, and OA was 72.5%, 73.8%, and 71.5%, respectively, and only the value under PM was greatly promoted to 83.3%. The promotion of PM on fine root (d < 1.0 mm) contributed to the decrease in RAD and RV. The promotion effect of OA on RLD (60.6%) was significantly higher than that of PM (21.2%) and SI (8.1%). In addition, RSA and RV under OA were also significantly increased by 69.57% and 25.37%, respectively. OA did not influence the root diameter component, and its promotion effect was for the root at the different soil layers and the root with different diameters (**Figure 8**). There was no significant difference in soil morphological traits between SI and CK. However, in the 0–10 cm soil layer, RLD for diameter < 0.5 mm was significantly improved by 37.30% under SI.

**Table 2:** Root morphological traits of cotton under the control (CK), plastic mulching (PM), straw interlayer (SI), and organic amendment (OA) treatments.

Treatments	Root morphological traits			
	RLD (cm/cm^3^)	RSA (cm^2^/cm^3^)	RV (%)	RAD (mm)
CK	0.099 ± 0.020 c	0.023 ± 0.009 b	0.067 ± 0.024 b	0.646 ± 0.033 a
PM	0.120 ± 0.019 b	0.022 ± 0.005 b	0.036 ± 0.011 c	0.539 ± 0.029 b
SI	0.107 ± 0.013 c	0.025 ± 0.007 b	0.054 ± 0.017 b	0.676 ± 0.022 a
OA	0.159 ± 0.042 a	0.039 ± 0.011 a	0.084 ± 0.020 a	0.703 ± 0.082 a

Values represent means ± SD. Different letters indicate the significant difference among treatments at p ≤ 0.05.

RLD, root length density; RSA, root surface area; RV, root volume; RAD, root average diameter.

### 3.3 Relationships between cotton root characteristics and soil environment

**Figure 9:**
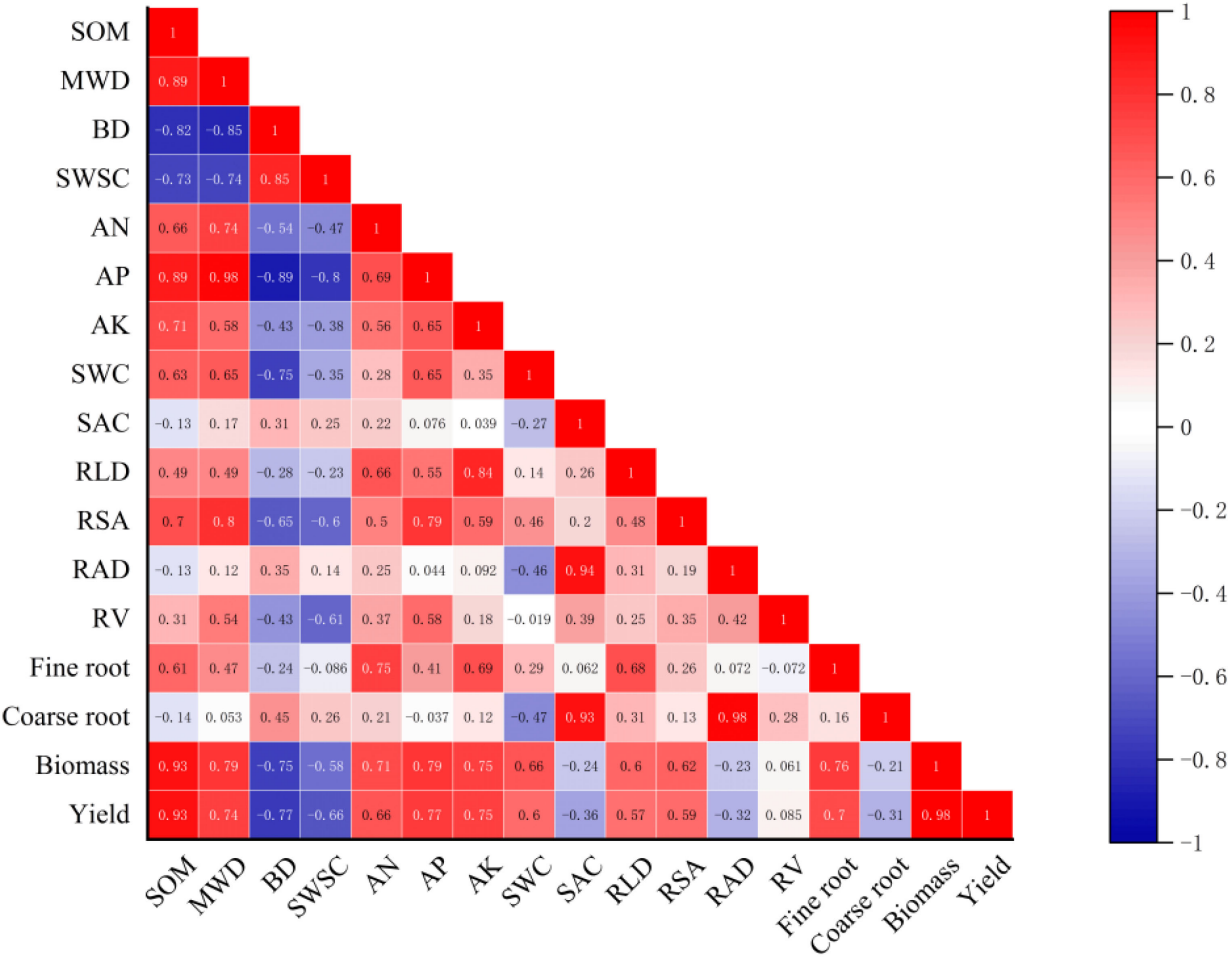
Relationships between cotton growth parameters and soil properties in 2021. SOM, soil organic matter; MWD, mean weight diameter; BD, bulk density; SWSC, soil water storage capacity; AN, alkali hydrolyzed nitrogen; AP, available phosphorus; AK, available potassium; SWC, soil water content; SAC, soil salt content; RLD, root length density; RSA, root surface area; RV, root volume; RAD, root average diameter. Values in layout indicate the correlation coefficient.

**Figure 10:**
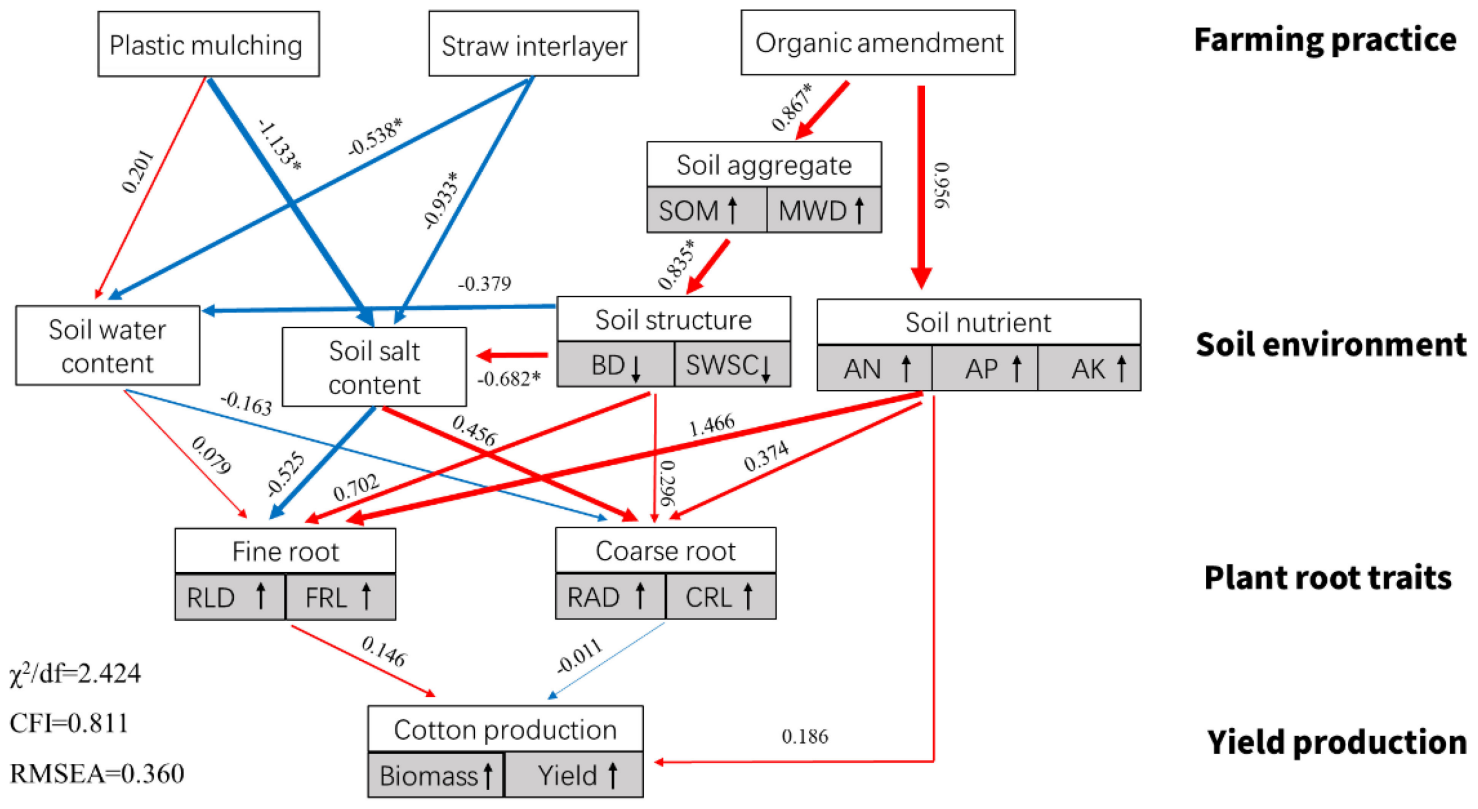
Structural equation model (SEM) reflecting the influence of soil properties on cotton root traits and yield production in 2021. Note: single-headed arrows represent the hypothesized direction of causation. Red and blue arrows represent the positive and negative relationships, respectively. The numbers on the arrows are normalized path coefficients. *indicates the degree of explanation with significance (p ≤ 0.05). The symbols ↑ and ↓ represent the positive and negative relationship between the related variables and their component, respectively. SOM, soil organic matter; MWD, mean weight diameter; BD, bulk density; SWSC, soil water storage capacity; AN, alkali hydrolyzed nitrogen; AP, available phosphorus; AK, available potassium; SWC, soil water content; SAC, soil salt content; RLD, root length density; FRL, root length for fine root (d < 0.1 mm); RAD, root average diameter; CRL, root length for coarse root (d > 0.1 mm).

Based on the field data in 2021, Pearson’s correlation analysis showed that the yield of cotton was strongly positively related to SOM, MWD, AP, and AK and negatively related to BD, SWSC, and SAC (**Figure 9**). In addition, cotton root morphological characteristics presented different influences on the growth of cotton. RLD and RSA were positively related to cotton biomass and yield, but they were negatively related to between cotton yield. Furthermore, a structural equation model was established to reflect the influence of farming practices on soil environment and cotton root characteristics (**Figure 10**). It showed that PM and SI directly influenced soil water and salt content, while OA improved soil structure, which had impacts on soil water and salt content. The cotton root characteristics were mostly determined by soil environment, including soil water, salt, structure, and nutrient factors. The fine root of cotton was positively related to soil water content and negatively related to soil salt content. However, the coarse root showed opposite relationships with soil water and salt content. Soil structure and nutrients represent the same positive relationship on both the cotton fine and coarse roots.

## 4 Discussion

### 4.1 Cotton root adaptive characteristics under the different farming practices

Water deficit and soil salinity are major limiting factors for crop production in saline lands. These conditions bind water to the soil matrix through adsorption, resulting in the osmotic stress and ion toxicity to plants (Tejada et al., 2006; Mahmood and Mohammad, 2020). In the Bohai coastal low plain, the groundwater level is shallow (0.8–2.0-m depth) and has a high salt content (15–40 g/L), and the ratio of precipitation/soil evaporation is approximately 1:3.5 (Zhang P. et al., 2014; Xia et al., 2019), which drives the irreversible accumulation of salt from saline groundwater to the soil surface. Salt distribution in soils is never uniform across an area; it varies with soil depths (Munns et al., 2020). Therefore, preventing upward salt capillary action was considered the most effective and water-saving method to control the root-zone salt content.

**Figure 11:**
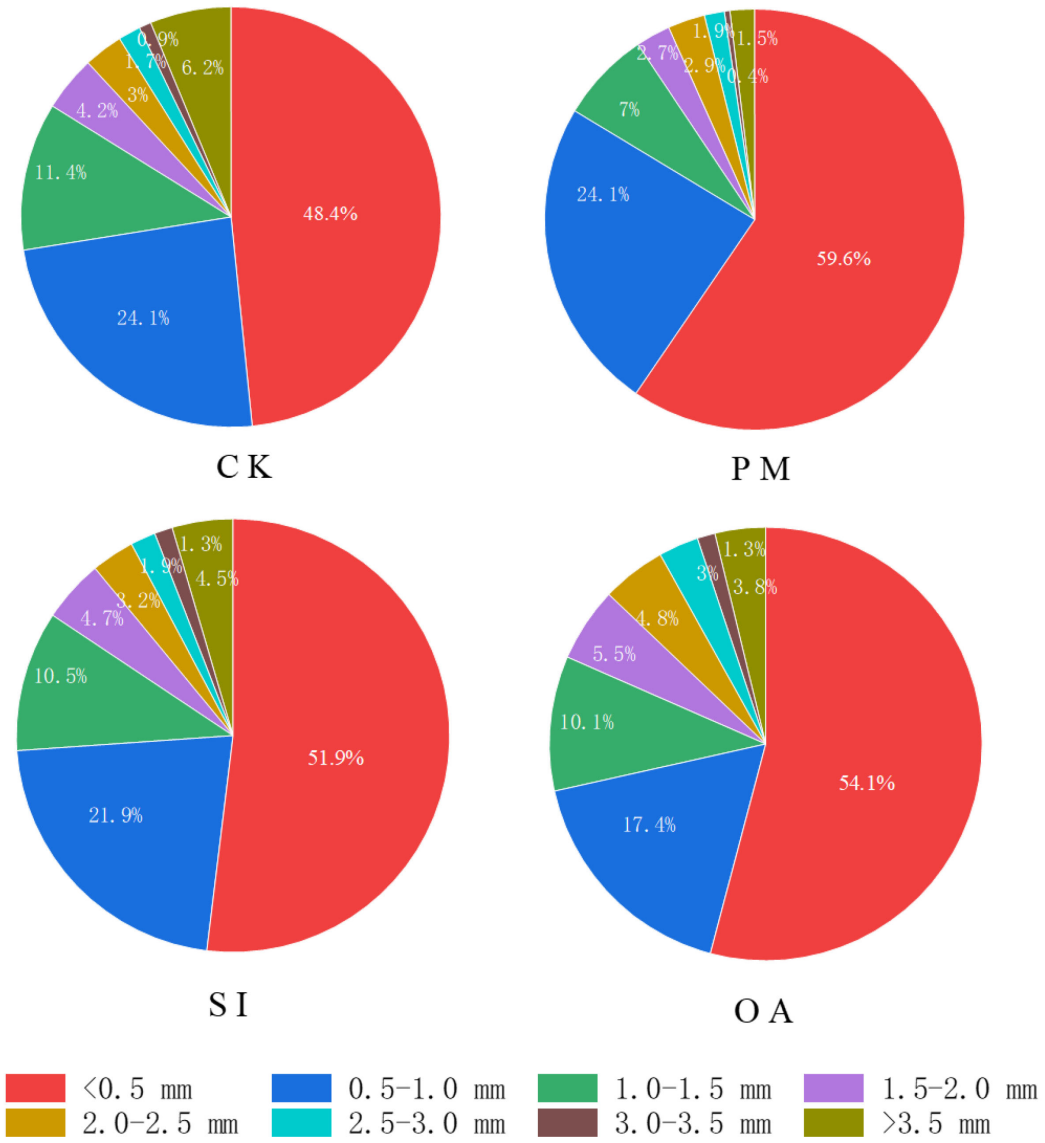
The percentage of different root diameters under the control (CK), plastic mulching (PM), straw interlayer (SI), and organic amendment (OA) in topsoil.

The results of this study indicate that PM, SI, and OA all effectively minimized soil salinization in coastal cotton farmland, which is consistent with the results of the previous studies of Dong (2012), Chavez and Siebe (2019), and Umidbek et al. (2024). Furthermore, these reclamation practices resulted in different soil water, structure, and nutrients. PM could inhibit soil evaporation and effectively control the salt stored at deep soil to avoid ion toxicity for plants (Ning et al., 2021; Chen et al., 2024). In this study, it was observed that compared with CK, more cotton roots were distributed in 0–0.2-m topsoil under PM (**Figure 7**). In addition, the proportion of root diameter composition in topsoil was significantly changed (**Figure 11**). The positive effect of PM on cotton root was mainly reflected in the stimulation of fine roots. Under the suitable soil environment created by PM, a large number of new roots developed, resulting in an overall increase in RLD and a decrease in RAD (**Table 2**). Since root foraging ability is largely determined by RLD and root system architecture (Shelden and Munns, 2023; Holz et al., 2024), it can be concluded that cotton preferentially allocates more carbohydrates to roots in soil zones with lower salinity and higher moisture to maintain growth and yield through modifications in root system architecture under PM. Capillary barriers could prevent, or at least minimize, the salt accumulation on the soil surface (Liu et al., 2022). However, in this study, it was observed that soil moisture above the buried straw layer was also significantly limited. Compared with CK, SI created a lower salinity but drought topsoil environment (**Figure 5**). Although SI improved 31.47% of cotton yield (**Figure 6**), there was no significant difference between SI and CK on root morphological characteristics, root distribution in the soil profile, and root diameter component. OA was one of the most common approaches to improve the soil quality (Tejada et al., 2013; Sastre Conde et al., 2015). Our results showed that OA substantially altered soil physical and chemical properties, creating a favorable soil environment characterized by low bulk density and large aggregate structure in the restored soil layer, which influenced soil salt dynamics. OA effectively controlled soil salts in the root zone, achieving soil desalination with the mechanism described in our previous study (Li et al., 2024b). Higher topsoil organic matter, increased nutrient content, and reduced salt concentration collectively promoted cotton root growth not only in the restored soil layer but also in deeper soil layers. As shown in **Figure 7**, RLD, RAS, and RV under OA were all higher than those under PM and SI at each soil layer. The SEM also indicated that there was no difference in the promotion effect of OA on the fine root and coarse root (**Figure 10**). Thus, we suggested that cotton plant positively responds to the OA-induced soil improvement by the collaboration of aboveground growth and root establishment.

### 4.2 The relationship between cotton root morphology and soil environment

The primary functions of root systems are to acquire water and nutrients from the soil and to anchor plants (Vanderborght et al., 2024). Roots are in direct contact with the soil environment and are greatly affected by stress, such as drought, salt, and flooding. Under salt stress, plant roots are the first to sense stress signals and produce corresponding physiological responses (Francisco et al., 2022). The ability of root systems to perceive and respond to stress mainly depends on their adaptation to the soil environment (Koevoets et al., 2016). Plants exhibit root plasticity and can modulate their root system architecture in response to their environment. A well-developed root system is fundamental to increasing crop yield. Thus, the morphology and physiological ecology of the root system have become key focuses and hot topics in root system research.

Under stressful environmental conditions, the morphological parameters of plant roots, such as root length, root surface area, and root volume, undergo significant changes that adversely affect plant growth. Generally, when soil salinity increases, the number of cotton roots decreases and root length shortens. In this study, the amelioration of saline land reflected the improvement of soil structure, nutrients, and moisture, and a decrease in soil salinity for partial soil (**Figure 2**). It was observed that roots tended to develop in areas with high soil moisture and low salinity. This variation in root distribution was important for the water absorption of the cotton plant. As reported by Bazihizina et al. (2009) and Shalhevet and Bernstein (1968), more than 70% of the total water uptake was from the lower saline zones under vertically or horizontally heterogeneous salinity. Water extraction is affected not only by the local salinities in the root zone but also by root distribution (Homaee and Schmidhalter, 2008; Nadia et al., 2012). It was also proposed that plant root was closely related to soil aggregates and the availability of water and salt in different soil aggregates (Vanderborght et al., 2024). Our study indicated that the growth and distribution of cotton roots were highly determined by the root-zone soil environment. However, cotton roots respond differently to the improvement of soil moisture, salinity, structure, and nutrients, as shown in the structural equation model (**Figure 10**). Notably, soil moisture and salinity showed the opposite effects on the growth of fine root (d < 1.0 mm) and coarse root (d > 1.0 mm). In summary, there were special physiological and molecular mechanisms underlying cotton’s response to heterogeneous root-zone soil environments. Plants could optimize the use of less salt, higher moisture, nutrients, and greater structure patches in the root zone soil, thereby enhancing the growth under heterogeneous saline conditions. We suggest that this root adaptation strategy also exists in other crop species. The results of this study can be applied to improve the root system architecture for crop production in saline land.

## 5 Conclusions

Farming practices involving PM, SI, and OA increased cotton yield in coastal saline land by 45.83%, 31.47%, and 57.58%, respectively, due to improvements in the soil environment. However, their effects on root-zone soil extended beyond merely reducing soil salinity. PM increased soil water; SI decreased soil water; OA had little effect on soil water, but improved MWD, BD, and soil nutrients. It was found that cotton yield was greatly determined by root establishment, especially the length of fine roots (d < 1.0 mm). Cotton root responses were primarily influenced by variations in soil conditions: soil water promoted RLD and the proportion of fine roots, counteracting the effects of salinity. Soil salt promoted the RAD and coarse root growth. However, the improved soil structure and nutrients would indiscriminately increase both the fine and coarse roots. Thus, it can be concluded that the composition of the cotton root diameter system is mainly determined by soil water and salt, rather than soil structure and nutrients. In coastal soil profiles, cotton plants can compensate for soil water and nutrient deficiencies and limited salt stress by optimizing root foraging in the most favorable soil layers, thereby alleviating salt toxicity under farming reclamation.

## Data Availability

The raw data supporting the conclusions of this article will be made available by the authors, without undue reservation.
